# Spontaneous Remission of Acute Myeloid Leukemia With Mutated *NPM1* Following SARS‐CoV‐2 Infection

**DOI:** 10.1155/crh/3373759

**Published:** 2026-07-24

**Authors:** Isabella Holmes, David Nai, Tamara Pikivaca, Praveena R. Solipuram, Anamarija M. Perry

**Affiliations:** ^1^ Department of Pathology, University of Michigan, Ann Arbor, Michigan, USA, umich.edu; ^2^ Department of Pathology, The University of Iowa, Iowa City, Iowa, USA, uiowa.edu; ^3^ Department of Pathology and Cytology, Clinical Hospital Centre Zagreb, Zagreb, Croatia, unizg.hr; ^4^ Division of Medical Oncology/Hematology, Rocky Mountain Cancer Centers, Thornton, Colorado, USA

**Keywords:** acute myeloid leukemia, *NPM1* mutation, spontaneous remission

## Abstract

Acute myeloid leukemia (AML) with *NPM1* mutation is the most common of the genetically defined AMLs in adults. Spontaneous remission (SR) of AML is very rare but well documented in the literature with < 40 reported cases in the last 25 years**.** SR of AML is usually short in duration, and most patients relapse in < 6 months**.** Causes of SR of AML are unclear, but most cases occurred in association with infection, including a few recently reported cases associated with SARS‐CoV‐2 infection. We report a unique case of a 77‐year‐old female with a 4‐year history of cytopenias prior to diagnosis of AML with mutated *NPM1*, concurrent with an infection by SARS‐CoV‐2. She subsequently experienced a SR of AML with loss of the *NPM1* mutation. After entering SR, the patient was initiated on treatment with subcutaneous azacitidine and venetoclax and has remained in remission for 44 months. Clinicians should be aware of this rare phenomenon and closely follow up these patients, as the SR of AML is usually of short duration.

## 1. Introduction

Acute myeloid leukemia (AML) with *NPM1* mutation is the most common of the genetically defined AMLs in adults, constituting nearly one‐third of AMLs overall and 50%–60% of AMLs with normal karyotype [1, 2]. It is defined by mutations in Exon 12 of the Chromosome 5 gene encoding nucleophosmin, a nucleolar protein which shuttles between the nucleus and cytoplasm and has many functions related to growth and proliferation [3]. AML with an *NPM1* mutation does not require a 20% blast count for diagnosis. According to the International Consensus Classification (ICC), the blast count required for diagnosis is ≥ 10% [4], while the 5^th^ Edition of the World Health Organization (WHO) Classification states that the blast count can be < 20% [5]. AML with *NPM1* mutation is associated with a relatively good prognosis after chemotherapy, with an 80% complete remission and 40% overall survival rate [6]. In new diagnoses above the age of 65, however, standard chemotherapy has a complete response rate of only 56% and a 1‐year overall survival rate of 36% [7]. Morphologically, this AML subtype skews toward the myelomonocytic and monocytic/monoblastic appearance of blasts. The bone marrow shows marked hypercellularity, and a quarter of cases exhibit multilineage dysplasia. AML with mutated *NPM1* frequently carries additional mutations, for example, in DNA methylation‐related genes (*DNMT3A*, *TET2*, and the *IDH* genes), *WT1*, *SRSF2*, and *FLT3* [8].

Spontaneous remission (SR) of AML is very rare but well documented. In the last 25 years, there have been < 40 cases reported in the English literature. Herein, we report a case of a 77‐year‐old female who was diagnosed with AML with mutated *NPM1*, concurrent with an infection by SARS‐CoV‐2. She subsequently experienced a SR of AML with loss of the *NPM1* mutation and has remained in remission for 44 months.

## 2. Case Report

The patient is a 77‐year‐old female with a 4‐year history of leukopenia with neutropenia and anemia prior to the diagnosis of AML. In this 4‐year period, her white blood cells (WBCs) ranged from 1.9 to 2.7 × 10^9^/L (ref. range: 3.7–11.1 × 10^9^/L), with neutrophils ranging from 0.1 to 1.6 × 10^9^/L (ref. range: 1.8–7.8 × 10^9^/L). Her hemoglobin (Hgb) fluctuated from 9.0 to 12.1 g/dL (ref. range: 11.9–16.3 g/dL). The patient was worked up 4 years before the diagnosis of AML for cytopenias; however, no definitive diagnosis of myeloid neoplasm was made. The workup included a myelodysplastic syndrome (MDS) fluorescence in situ hybridization (FISH) panel on peripheral blood, which was negative, and a bone marrow biopsy with no increase in blasts, unremarkable flow cytometry, and normal female karyotype by conventional cytogenetics. The patient’s past medical history was remarkable for arthritis with positive antinuclear antibody. She took no medications other than vitamin supplements.

**FIGURE 1:**
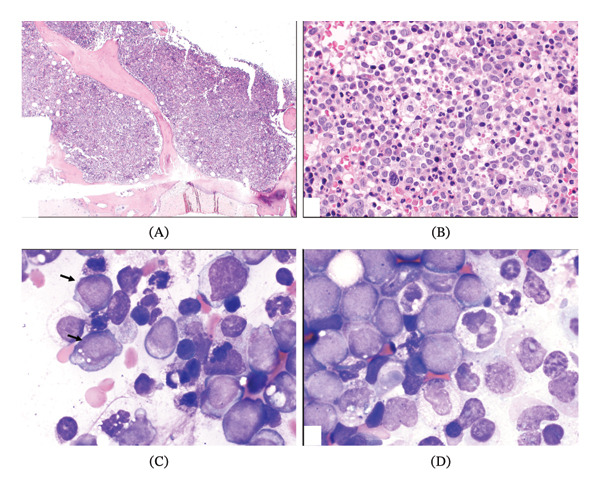
Diagnostic bone marrow biopsy showing markedly hypercellular marrow by hematoxylin and eosin (H&E) staining (A; original magnification × 20), with numerous immature cells, consistent with blasts (B; original magnification × 200). Wright–Giemsa staining of aspirate smears shows increased myeloid blasts (arrows, C; oil, original magnification × 1000) and prominent dysplasia in maturing myeloid cells (D; oil, original magnification × 1000).

The patient developed SARS‐CoV‐2 infection and presented to the emergency department with several days of productive cough, congestion, fevers, chills, and vomiting. She was not vaccinated for SARS‐CoV‐2. On presentation, she was febrile with tachycardia and hypoxia, and on auscultation, coarse breath sounds were heard throughout the lungs. The patient tested positive for SARS‐CoV‐2, and chest X‐ray showed multifocal opacities consistent with COVID‐19 pneumonia. She was given intravenous fluids and acetaminophen and was placed on oxygen (2 L via nasal cannula). Her condition improved, and she was discharged home on 2 L oxygen via nasal cannula. Shortly after COVID‐19 infection, the patient presented to the hospital with a rash, worsening pancytopenia, and elevated liver enzymes. Complete blood count showed Hgb of 7.7 g/dL, WBC of 3.2 × 10^9^/L, and platelets of 56 × 10^9^/L (ref. range: 150–400 × 10^9^/L). Her lactate dehydrogenase (LDH) was elevated at 725 UI/dL (ref. range: 105–333 UI/dL). Bone marrow biopsy was performed and was markedly hypercellular for age (90% cellularity) with 40% blasts by differential count (Figures 1A and B). The blasts had a high nuclear to cytoplasmic ratio, fine chromatin, and occasional prominent nucleoli (Figure 1C). Flow cytometry of the marrow aspirate showed < 1% CD34+/CD117+ myeloid blasts and approximately 10% CD34−/CD117+ cells, with apparently normal myeloid antigen expression. Immunohistochemical stains, performed on the bone marrow core, confirmed that blasts were CD34‐/CD117+. Background maturing myeloid cells showed dysplasia with pelgeroid forms, nuclear/cytoplasmic asynchrony, and hypogranulation (Figure 1D). Megakaryocytes also showed dysplasia with small hypolobated forms and separate nuclear lobes, while erythroid precursors displayed abnormal maturation with nuclear budding and megaloblastoid morphology. Conventional cytogenetic analysis showed a normal female karyotype. Molecular studies (next‐generation sequencing) found an R132S (c.394C > A) *IDH1* mutation at 45% variant allele frequency (VAF) and an *NPM1* frameshift mutation (p.W288Cfs∗12, a four‐nucleotide CCTG insertion) at 19% VAF. There was no evidence of *FLT3-*ITD or ‐TKD mutations. The patient was diagnosed with AML with a mutated *NPM1*. Due to poor performance status and anticipating that she would not tolerate chemotherapy well, she was discharged to hospice. Aside from transfusion of blood products, the patient did not receive any AML‐directed treatment.

**FIGURE 2:**
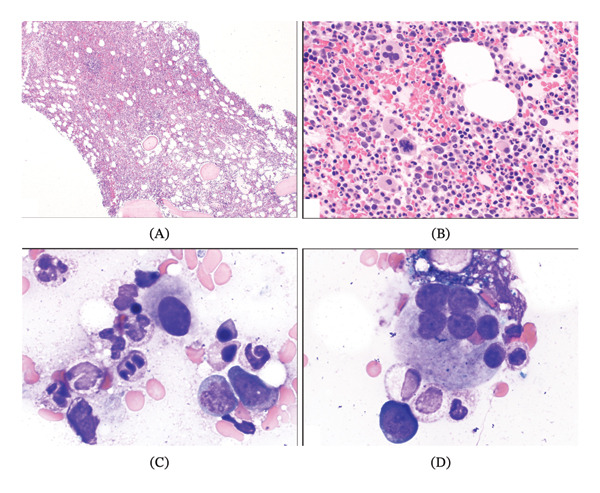
Bone marrow biopsy showing spontaneous regression of acute myeloid leukemia. Bone marrow is hypercellular by H&E staining (A; original magnification × 20) and shows myeloid predominance with maturation and dysplastic megakaryocytes (B; original magnification × 200). Wright–Giemsa staining of aspirate smears shows prominent dysplasia in all myeloid and erythroid lineages and megakaryocytes (C and D; oil, original magnification × 1000).

Over the course of approximately 4 months, the patient experienced an improvement in her clinical picture and performance status and became transfusion independent. Moreover, four months after the initial diagnosis of AML, the patients’ blood counts improved with Hgb of 11.7 g/dL, WBCs of 2.4 × 10^9^/L, and platelets of 169 × 10^9^/L. Given this clinical improvement, the patient was initiated on treatment with subcutaneous azacitidine (130 mg s.c. for 5 days) and venetoclax (100 mg p.o. q.d on Days 1–5, every 28 days). Three days after the start of chemotherapy, the repeat bone marrow biopsy was performed. Bone marrow was still hypercellular for age (80% cellularity) and showed increased myeloid–erythroid ratio but no increase in blasts (Figures 2A and B). Differential count showed 1.2% promyelocytes, 62.4% neutrophil precursors, 11.4% erythroid precursors, 16.0% lymphocytes, 4.0% monocytes, 0.4% plasma cells, and 0.2% basophils. Dysplasia was still present in all hematopoietic lineages (Figures 2C and D). The concurrent flow cytometry showed < 1% CD34+/CD117+ myeloid blasts and 2% CD34−/CD117+ immature myeloid precursors. Immunohistochemical stains, performed on the bone marrow core, showed no increase in CD34‐negative or CD117‐positive precursors. Next‐generation sequencing showed persistent *IDH1* mutation, but no evidence of *NPM1* mutation. Findings were consistent with persistent myeloid neoplasm with no increase in blasts, best classified as MDS.

The patient completed 11 cycles of azacitidine and venetoclax. A bone marrow biopsy, performed approximately 19 months after initial AML diagnosis, showed 3% blasts, normal female karyotype, persistence of *IDH1* mutation, and no evidence of *NPM1* mutation. The patient continued with venetoclax‐only treatment, and azacitidine was discontinued due to low platelet counts. On the last follow‐up, 44 months after the initial diagnosis of AML, the patient’s counts remained stable; she was asymptomatic and reported feeling well.

## 3. Discussion

We report an unusual case of sustained SR of AML with an *NPM1* mutation coinciding with COVID‐19 illness. SR of AML is a rare but well‐described phenomenon with 36 cases reported in the English literature in the last 25 years. A subset of these cases was compiled and reviewed by Rashidi et al. [9–25]. There are also a few reports of SR of congenital AML, which are not included in this review. Median age of reported patients was 56 years (range: 12–83 years), with a male: female ratio of 1.1. In the majority of cases (68%), the patients had an associated infection with various pathogens, most frequently pneumonia or bacteremia. Follow‐up data were available for 35 patients, and in 24/35 (68%) patients, AML relapsed, with a median time to relapse of 3.5 months (range: 0.5–14 months). Moreover, 83% of patients relapsed in 6 months or less. Few patients with longstanding SR were reported, with one patient being in remission for 10 years.

Owing to the rarity of SR in AML, no strong cytogenetic or molecular associations are evident [9]. In addition to our case, there have been 6 cases of SR of AML with *NPM1* reported in the literature [14, 21, 22, 25]. Clinical, pathologic, and genetic findings of these patients are summarized in Table 1. There were 2 males and 4 females, with a median age of 65.6 years (range: 24–74 years). Four patients presented with infection, including one who had SARS‐CoV‐2. Conventional cytogenetic analysis was available in 5 patients, and 3 had a normal karyotype, one had del(15q), and one had a complex karyotype. In 5/6 patients, AML relapsed, with a median time to relapse of 2 months (range: 1.5–12 months). In addition to *NPM1* mutation, one patient had *FLT3*‐ITD mutation, one had *TP53* mutation, and the patient reported by Grunwald et al. [21] had seven additional mutations. Interestingly, during SR of AML, the *NPM1* mutation in this patient disappeared, while other mutations persisted; at relapse, the *NPM1* mutation reappeared. This is similar to our patient, who has had a persistent *IDH1* mutation while *NPM1* disappeared. Of note, in most case reports, only targeted mutational analyses were performed and not comprehensive sequencing.

**TABLE 1:** Clinical, pathologic, and genetic findings of reported cases of acute myeloid leukemia with mutated *NPM1* that spontaneously regressed.

Case	Age/sex	Blast % at diagnosis in the BM	Blast immunophenotype	Karyotype	Molecular findings	Other conditions coinciding with the SR of AML	Duration of spontaneous remission	Therapy given at relapse
Current case	77/F	40%	CD34+, CD117+	Normal female	Positive: *NPM1* frameshift mutation (p.W288Cfs^∗^12, a four‐nucleotide CCTG insertion) *IDH1* ^∗^	SARS‐CoV‐2 infection	44 months	Still in remission
Camus et al. [14]	24/F	58%	NA	NA	Positive: *NPM1*c.861_862insTGTC Negative: *CEBPA*, *FLT3*‐ITD, and ‐TKD	Febrile polyarthralgia; broad spectrum antibiotics administered	2 months	NA
Camus et al.	33/M	10.5%	CD15+, CD68+, CD117+, CD34‐, CD163‐, MPO‐	Normal male	Positive: *NPM1* (an insertion of TATG) Negative: *CEBPA*, *FLT3*‐ITD and ‐TKD, *MLL*	Fever, sepsis; antibiotics (piperacillin/tazobactam) administered	1.5 months	3 courses of high‐dose cytarabine
Camus et al.	74/F	55% blasts and promonocytes	CD13+, CD14+, CD33+, CD64+, CD117+	Normal female	Positive: *NPM1* (duplication of TCTG) Negative: *CEBPA*, *FLT3*‐ITD and ‐TKD, *MLL*	Fever, pneumonia; antibiotics (amoxicillin/clavulanic acid) administered	2 months	NA
^∗∗^Vachhani et al. [22]	73/F	64% blasts	CD13+, CD15+, CD33+, CD58+, CD117+, HAL‐DR+	del(15q)	Positive: *NPM1* *FLT3-*ITD mutation	None	1.5 months	Induction: cytarabine (100 mg/m^2^ once daily x 7 days) and daunorubicin (45 mg/m^2^ once daily x 3 days) Consolidation: 2 cycles of high‐dose cytarabine (1.5 g/m^2^ once daily x 6 days)
Grunwald et al. [21]	72/M	85%	NA	Normal male	Positive: *NPM1* c.860_863dup p. (Trp288 fs^∗^12) *TET2* c.3954+1G > A p.?; *PRPF8*#c.6966G > T p. (Glu2322Asp) *RUNX1* c.990C > A p. (Phe330Leu) *NRAS* c.35G > A p.(Gly12Asp) *KRAS* c.436G > A p. (Ala146Thr) *U2AF1* c.100T > G p. (Ser34Ala) *NF1* c.2375T > A p. (Leu792His)	None	12 months	No therapy
Qi [25]	59/F	68%	MPO, CD13, CD33, CD34, CD117, CD38, and CD123	46–50,XX, +4, +6, +8, +21/46,XX	Positive: *NPM1* (25%) and *TP53* (29%) mutation	SARS‐CoV‐2 infection	3 months^∗∗∗^	Induction: azacytidine (75 mg/m^2^/dose, days 1–7) and venetoclax (VEN 100/dose, Days 1–7) Consolidation: 2 cycles ‐azacytidine (75 mg/m^2^/dose, days 1–28) and venetoclax (400 mg/dose, Days 1–28)

Abbreviations: AML, acute myeloid leukemia; BM, bone marrow; MPO, myeloperoxidase; NA, not available; SR, spontaneous remission.

^∗^ *IDH1* mutation persisted after SR of AML.

^∗∗^The patient entered SR after the first relapse of treated AML.

^∗∗∗^The patient was in remission at 3‐month follow‐up.

The vast majority of reported SRs of AML occurred following a fever (91%), which was caused most frequently by pneumonia or bacteremia. Around the turn of the 20^th^ century, an American physician named William Coley theorized that inflammation was an effective anticancer mechanism. By injecting preparations of heat‐killed *Streptococcus pyogenes* and *Serratia marcescens* (so‐called “Coley’s toxins”) into several patients’ sarcomas, he reported anecdotally that the patients survived much longer than was typical for their diagnoses [26]. While this therapy never caught on, it is remarkable for probing the link between inflammation, including that caused by the immune system’s anti‐infectious response, and the course of malignancy. Most of the literature focuses on inflammation being the key driver in eliminating malignant cells [9, 11, 14, 27]. It has been postulated that the increased expression of heat shock proteins (HSPs) by the neoplastic cells is followed by tumor cell elimination by T‐cells that have receptors for the HSP epitopes. Another theory is that the cytokine release during febrile infections upregulates MHC Class I–associated tumor antigens, which co‐stimulate tumor antigens and T‐cells. In addition, it has been thought that febrile temperatures induce the maturation of dendritic cells through the elevation of intracellular levels of HSP90, and thus elimination of tumor cells [28].

Acute leukemia patients are particularly vulnerable to SARS‐CoV‐2 infection, with a high‐risk of severe COVID‐19 illness and death, generally necessitating a delay in leukemia‐oriented treatment and often causing a cytokine storm [29]. This cytokine storm results in the massive release of inflammatory mediators that cause downstream activation of the immune response, leading to the recruitment of leukocytes and endothelial damage [30]. Some of these include tumor necrosis factor (TNF); interferons such as IFNα, ‐β, and ‐γ; the interleukins IL‐1, IL‐2, IL‐6, and IL‐8; chemokines CXCL1, CXCL2, and CCL2; Macrophage inflammatory protein 1 (MIP1*α*); and Monocyte chemoattractant protein 1 (MCP‐1) [31, 32]. The virus is known to activate toll‐like and RIG‐I–like receptors (TLR and RLR) as well as to induce the canonical NF‐κB pathway, which has profound effects on the regulation of apoptosis and the immune response to pathogens [33, 34]. Production of hypoxia‐inducible factor (HIF)–1*α*, a key proinflammatory regulator of cell proliferation and angiogenesis, is also involved in the virus’ pathogenesis [35]. These inflammatory substances and pathways show extensive overlap with factors implicated in an antitumor effect, providing a possible mechanism for our patient’s SR. It is additionally posited that activation of these innate antitumor pathways causes natural killer cell activation and sets in motion a T‐cell response against tumor antigens [36].

There have been multiple reports of SR of hematologic malignancies following SARS‐CoV‐2 infection, including remissions of low‐ and high‐grade B‐cell non‐Hodgkin lymphoma (NHL), T‐ and NK‐NHL, as well as classic Hodgkin lymphoma [37–40]. Also, several reports of SR of myeloid neoplasms were reported, including chronic myeloid leukemia and AML [16–18, 25, 41]. Barkhordar et al. [16] described a case of a 57‐year‐old female with AML with 11q23/*KMT2A* abnormality who was admitted to intensive care and received remdesivir and dexamethasone for severe COVID‐19 disease. She attained a complete molecular and cytogenetic SR but recurred 8 months later. Kandeel et al. [17] report a case of a 63‐year‐old female with AML with monocytic differentiation who attained a SR after recovering from moderate COVID‐19 illness. The patient was in remission for 1 year at the time of the case report publication. Penuela et al. [18] reported a case of a 25‐year‐old female with acute myelomonocytic leukemia, normal karyotype, and a wild‐type *FLT3* ITD‐TKD. The patient developed COVID‐19 prior to initiation of antineoplastic chemotherapy and was given low‐dose dexamethasone and colchicine. She recovered from COVID‐19 and her hemogram returned to normal, prompting the bone marrow biopsy, which showed no evidence of AML. The patient relapsed after 5 months of follow‐up. Our patient, in comparison, had a history of anemia and neutropenia for several years prior to AML diagnosis. No bone marrow biopsy was performed to prove it, but the patient likely had MDS and eventually progressed to AML with a mutated *NPM1*. Although the SR of AML coincided with COVID‐19 disease, her remarkably long (44 months) remission is likely attributed to azacitidine and venetoclax maintenance therapy. The bone marrow biopsy, performed 19 months after AML diagnosis, still showed evidence of dysplasia with an *IDH1* mutation, but the counts remained stable, and the patient reported feeling well. Our case is somewhat similar to that reported by Qi et al. [25] of a 59‐year‐old female with AML with mutated *NPM1* with a complex karyotype and *TP53* mutation. This patient had partial SR after SARS‐CoV‐2 infection, with bone marrow blast reduction from 68% to 13%. She was then given azacitidine and venetoclax, and after induction and 2 cycles of consolidation, the patient was in complete remission, with a normal karyotype and no mutations. Only a short follow‐up (3 months) was available for this patient. The combination of azacitidine and venetoclax is an efficacious and relatively well‐tolerated treatment for both AML and MDS in elderly patients, as well as a good option for maintenance therapy in AML following intensive or low‐intensity induction [42–44].

## 4. Conclusion

SR of AML is a very rare but well‐documented phenomenon. We report a unique case of a 77‐year‐old female with AML with mutated *NPM1,* diagnosed concurrent within SARS‐CoV‐2 infection, who experienced disappearance of blasts and *NPM1* mutation without therapy. Given the long history of low blood counts that preceded AML and persistent bone marrow dysplasia, the patient was given azacitidine and venetoclax maintenance therapy and has remained in remission for 44 months with stable blood counts. Causes of SR of AML are unclear, but most reported cases occurred after an infection, including COVID‐19 illness, and remission was relatively of short duration (< 6 months in most patients). Clinicians should be aware of this rare phenomenon, and our case illustrates a potentially successful maintenance therapy after SR.

## Funding

No funding was received for this study.

## Disclosure

This patient was recruited in 2022 in Colorado, USA. All authors have read and approved the final version of the manuscript.

## Ethics Statement

Dr. Anamarija Perry had full access to all of the data in this study and takes complete responsibility for the integrity of the data and the accuracy of the data analysis.

## Consent

No written consent has been obtained from the patients as there are no patient identifiable data included in this case report.

## Conflicts of Interest

The authors declare no conflicts of interest.

## Data Availability

The data that support the findings of this study are available on request from the corresponding author. The data are not publicly available due to privacy or ethical restrictions.
